# Dystonia caused by ANO3 variants is due to attenuated Ca^2+^ influx by ORAI1

**DOI:** 10.1186/s12916-024-03839-5

**Published:** 2025-01-07

**Authors:** Jiraporn Ousingsawat, Khaoula Talbi, Hilario Gómez-Martín, Anne Koy, Alberto Fernández-Jaén, Hasan Tekgül, Esra Serdaroğlu, Juan Darío Ortigoza-Escobar, Rainer Schreiber, Karl Kunzelmann

**Affiliations:** 1 Physiological Institute, University of Regensburg, University Street 31, 93053 Regensburg, Germany; 2 Pediatric Neurology Unit, Department of Pediatrics, Hospital Universitario de Salamanca, 37007 Castillay , Leon, Spain; 3 Centre for Rare Diseases, Faculty of Medicineand , University Hospital Cologne, University of Cologne, 50931 Cologne, Germany; 4 Department of Pediatrics, Faculty of Medicine and University, Hospital Cologne, University of Cologne, 50931 Cologne, Germany; 5 Department of Pediatric Neurology, Hospital Universitario Quirónsalud, 28223 Pozuelo de Alarcón, Madrid, Spain; 6 School of Medicine, Universidad Europea De Madrid, 28670 Villaviciosa de Odón, Madrid, Spain; 7 Division of Pediatric Neurology, Ege Children’s Hospital, Ege University Medical School, 35100 Bornova, Izmir, Turkey; 8 Department of Pediatric Neurology, Gazi University, Emniyet, Ankara Yenimahalle, 06560 Turkey; 9 Movement Disorders Unit, Pediatric Neurology Department, Institut de Recerca Hospital Sant Joan de Déu Barcelona, Barcelona, Spain; 10 U-703 Centre for Biomedical Research On Rare Diseases (CIBER-ER) Instituto de Salud Carlos III, Barcelona, Spain; 11 European Reference Network for Rare Neurological Diseases (ERN-RND, Barcelona, Spain

**Keywords:** Dystonia, TMEM16C, Anoctamin 3, ANO3, Ca^2+^ signaling, K^+^ channels

## Abstract

**Background:**

Dystonia is a common neurological hyperkinetic movement disorder that can be caused by mutations in anoctamin 3 (ANO3, TMEM16C), a phospholipid scramblase and ion channel. We previously reported patients that were heterozygous for the ANO3 variants S651N, V561L, A599D and S651N, which cause dystonia by unknown mechanisms.

**Methods:**

We applied electrophysiology, Ca^2+^ measurements and cell biological methods to analyze the molecular mechanisms that lead to aberrant intracellular Ca^2+^ signals and defective activation of K^+^ channels in patients heterozygous for the ANO3 variants.

**Results:**

Upon expression, emptying of the endoplasmic reticulum Ca^2+^ store (store release) and particularly store-operated Ca^2+^ entry (SOCE) were strongly inhibited, leading to impaired activation of K_Ca3.1_ (KCNN) K^+^ channels, but not of Na^+^-activated K^+^ channels (K_Na_; SLO2). The data provide evidence for a strongly impaired expression of store-operated ORAI1 Ca^2+^ influx channels in the plasma membrane of cells expressing ANO3 variants.

**Conclusions:**

Dysregulated Ca^2+^ signaling by ANO3 variants may impair the activation of K^+^ channels in striatal neurons of the brain, thereby causing dystonia. Furthermore, the data provide a first indication of a possible regulation of protein expression in the plasma membrane by ANO3, as has been described for other anoctamins.

**Supplementary Information:**

The online version contains supplementary material available at 10.1186/s12916-024-03839-5.

## Background

Parkinson's disease and dystonia are the two most common movement disorders which are related to defective intracellular Ca^2+^ signaling [1, 2]. Among the plethora of regulatory proteins, intracellular Ca^2+^ signals are shaped by anoctamins, a family of ten different Ca^2+^-activated ion channels and phospholipid scramblases that include ANO3 (anoctamin 3, TMEM16C) [3–5]. ANO3 is expressed in the brain and is particularly abundant in basal ganglia (https://www.proteinatlas.org/ENSG00000134343-ANO3/brain.). Basal ganglia regulate voluntary motor movements and are required for procedural learning, including the striatum as a main functional component. We previously reported four patients who are heterozygous for four different ANO3 variants (V561L, S651N, A599D and S651N), which cause dominant forms of craniocervical dystonia [6].

In brief, the patients were confirmed to express de novo heterozygous variants in the ANO3 gene. While three patients had an early-onset dystonia or an early-onset dyskinetic encephalopathy phenotype, another patient demonstrated a pronounced paroxysmal dystonia phenotype. Three patients showed diverse degrees of developmental delay accompanied by mild to severe intellectual and developmental disabilities. Bowel and urinary incontinence were observed in all three patients carrying the variants V561L, S651N, or A599D. The patient with the ANO3 variant S116L had no baseline movement disorder or any developmental problems, apart from paroxysmal dystonia in the lower limbs and a mild intellectual disability. Remarkably, functional in vitro analysis of ANO3-S116L showed only minor deviations from the normal wild-type ANO3 function, supporting the role of mutated ANO3 for the clinical phenotype. A detailed analysis of the phenotypes is described in our previous report [6].

In addition to the clinical phenotype, molecular aspects such as impaired intracellular Ca^2+^ signaling, enhanced cell death and defective potassium (K^+^) ion channel regulation by ANO3 variants were assessed in our previous report [6]. We concluded that expression of these disease-causing ANO3 variants in brain cells attenuate intracellular Ca^2+^ signals, which are controlled by G-protein coupled hormone receptors. However, the precise molecular mechanism responsible for attenuated Ca^2+^ signaling remained obscure. In the present study we report that ANO3 variants cause a reduced expression of the Ca^2+^ influx channel ORAI1 in the plasma membrane, which leads to attenuated filling of the endoplasmic reticulum (ER) Ca^2+^ store. Impaired Ca^2+^ signaling could compromise K^+^ channel activation, possibly leading to hyperexcitability in brain striatal cells and motor dysfunction.

## Methods

### Cell culture, cloning, transfection, and immunofluorescence

Human fibroblasts were grown a described previously [6]. Human embryonic Kidney 293 T (HEK293T) cells were cultured in DMEM with 10% FBS. HEK293T cells were transfected using standard protocols for Lipofectamine 3000 (Thermo Fisher Scientific, Germany). All experiments were performed 48–72 h after transfection. Generation of expression plasmids for the ANO3 variants A599D, S651N, V561L, and S116L was described in our previous report [6]. For patch clamp experiments and intracellular calcium measurements, ANO3-cDNA was subcloned into the bicistronic vector pIRES1-CD8. Immunofluorescence of overexpressed ORAI1 was performed with a mouse monoclonal anti-hORAI1 #168 and a donkey anti-mouse Alexa 488 (Sigma-Aldrich, Taufkirchen, Germany).

### Measurement of intracellular Ca^2+^ concentrations

Measurements of cytosolic Ca^2+^ were performed as described recently [7]. In brief, cells were loaded with 2 µM Fura-2, AM (BIOZOL, Eching, Germany) in OptiMEM (Gibco, Thermo Fisher, Scientific, Waltham, MA 02451, USA) with 0.02% Pluronic F-127 (Invitrogen, Thermo Fisher, Scientific, Waltham, MA 02451, USA) in Ringer solution (mmol/l: NaCl 145; KH_2_PO_4_ 0,4; K_2_HPO_4_ 1,6; Glucose 5; MgCl_2_ 1; Ca^2+^-Gluconate 1,3) for 1 h at room temperature. Fluorescence was detected in cells perfused with Ringer’s solution at 37 °C using an inverted microscope (Axiovert S100, Zeiss, Germany) and a high-speed polychromator system (VisiChrome, Puchheim, Germany). Fura-2 was excited at 340/380 nm, and the emission was recorded between 470 and 550 nm using a CCD-camera (CoolSnap HQ, Visitron Systems, Germany). [Ca^2+^]_*i*_ was calculated from the 340/380 nm fluorescence ratio after background subtraction. The formula used to calculate [Ca^2+^]_*i*_ was [Ca^2+^]_*i*_ = *Kd* x (*R*-*R*_min_)/(*R*_max_-*R*) x (S_f2_/S_b2_), where *R* is the observed fluorescence ratio. The values *R*_max_ and *R*_min_ (maximum and minimum ratios) and the constant S_f2_/S_b2_ (fluorescence of free and Ca^2+^-bound Fura-2 at 380 nm) were calculated using 2 µmol/liter ionomycin (Biomol GmbH, Hamburg, Germany) and 5 mmol/liter EGTA to equilibrate intracellular and extracellular Ca^2+^ in intact Fura-2-loaded cells. The dissociation constant for the Fura-2•Ca^2+^ complex was taken as 224 nmol/liter [8]. Control of the experiment, imaging acquisition, and data analysis were done with the software package Meta-Fluor (Universal Imaging, USA).

### Patch clamping

Cells were patch clamped when grown on fibronectin-coated glass coverslips at 37 °C. Patch pipettes were filled with a cytosolic-like solution containing (in mM): KCl 30, K-Gluconate 95, NaH_2_PO_4_ 1.2, Na_2_HPO_4_ 4.8, EGTA 1, Ca-Gluconate 0.758, MgCl_2_ 1.03, D-Glucose 5, ATP 3; pH 7.2. The intracellular (pipette) Ca^2+^ activity was 0.1 µM. The bath was perfused continuously with Ringer’s solution (in mM): NaCl 145, KH_2_PO_4_ 0.4, K_2_HPO_4_ 1.6, Glucose 5, MgCl_2_ 1, and Ca-Gluconate 1.3) at a rate of 6 mL/min. Patch pipettes had an input resistance of 2–5 MΩ and whole cell currents were corrected for serial resistance. The current voltage (I/V) relationships were determined by pulsing from the holding potential of −100 mV to test potentials between −100 and + 100 mV, increasing in 20 mV increments. Currents were recorded using the EPC-9 computer-controlled amplifier, PULSE software (HEKA), and Chart software (AD Instruments). The current density was calculated by dividing whole cell currents by cell capacitance.

### Cell death assays, flow cytometry

Cells were collected using accutase (Capricorn Scientific, Ebsdorfergrund, Germany), washed with cold Dulbecco’s PBS (DPBS), and centrifuged at 500 g and 4 °C for 10 min. Subsequently, cells were resuspended in 100 μL annexin binding buffer containing 5 μL annexin V-FITC and 2.5 μL 7-aminoactinomycin D (7-AAD; BioLegend, Koblenz, Germany). Cells were incubated with 10 μM ionomycin for 20 min. Reactions were stopped by adding 400 μL of DPBS, and cells were analyzed immediately. Fluorescence-activated cell sorting (FACS) analyses were performed in Annexin V standard binding buffer (BioLegend, San Diego, CA, USA) containing 10 mM Hepes, 140 mM NaCl, and 2.5 mM CaCl. For each measurement, at least 10,000 cells were analyzed by flow cytometry at 37 °C (BD AccuriTM C6, St. Ives, UK) 7-AAD, a non-permeant dye, was used to identify cells with plasma membrane leakage. Fibroblasts were labelled by propidium iodide uptake.

### Biotinylation

Biotinylation was described in our previous report [9]. After biotinylation for 30 min at 4 °C, cells were collected and lysed in 0.5% NonidetP40 lysis buffer. Proteins were separated by 8.5% SDS-PAGE and transferred to a PVDF membrane. The membrane was blocked with 5%NFM/TBS-T or 5%NFM/PBS-T for 1 h and incubated overnight at 4 °C with mouse anti-ORAI1 (diluted 1:1000 in 1% BSA/TBS-T; Sigma, Taufkirchen, Germany). Immunoreactive signals were detected using the SuperSignal chemiluminescence substrate (Pierce, Waltham, USA).

### Statistical analysis

Statistical analysis was performed using Student’s t-test (for paired or unpaired samples as appropriate) or ANOVA. A value of *p* < 0.05 was accepted as a significant difference. Data are reported as means ± SEM.

## Results

**Fig. 1:**
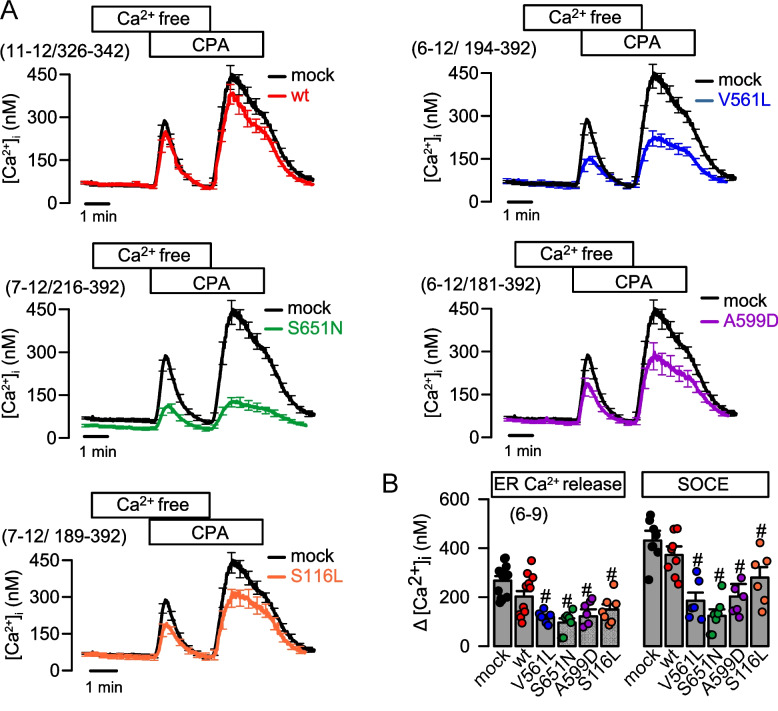
ANO3 variants reduce ER store filling and store-operated Ca^2+^ influx*.* **A** Measurement of [Ca^2+^]_i_ by Fura2 in mock-transfected HEK293 cells and HEK293 cells expressing wtANO3 (wt), or the ANO3 variants V561L, S651N, A599D, or S116L. Removal of extracellular Ca^2+^ by a Ca^2+^-free Ringer-like solution (Ca^2+^-free) and application of the SERCA-inhibitor cyclopiazonic acid (CPA, 10 µM). Subsequent replacement of Ca^2+^ free solution by Ringer solution. **B** Summary of changes in [Ca^2+^]_i_ induced by CPA (store emptying; Store) and removal of Ca^2+^ free solution (Ca^2+^ influx; SOCE). Mean ± SEM (number of experiments). ^#^significant difference when compared to mock (*p* < 0.05; ANOVA)

In our previous study, we determined intracellular Ca^2+^ concentrations ([Ca^2+^]_i_) in skin fibroblasts isolated from patients with dystonia and in HEK293 cells overexpressing dystonia-causing ANO3 variants (V561L, S651N, A599D, and S116L). We showed that all variants inhibited increase in intracellular Ca^2+^ due to stimulation of purinergic receptors with ATP, but the mechanism for this inhibition remained obscure [6]. Because the mechanisms for abrogated intracellular Ca^2+^ signaling remain obscure, we examined whether ANO3 variants affect filling of the ER Ca^2+^ store and/or Ca^2+^ influx through store-operated ORAI1 Ca^2+^ entry channels (SOCE). This was done by using Fura2 as Ca^2+^ fluorophore and cyclopiazonic acid (CPA), a reversible inhibitor of the sarcoplasmic endoplasmic reticulum Ca^2+^ ATPase (SERCA), which pumps Ca^2+^ back into the ER. CPA induced a complete emptying of the ER Ca^2+^ store in the presence of an extracellular Ca^2+^ free buffer, causing a transient rise in intracellular Ca^2+^ which is a measure of the store filling (Fig. 1A). Removal of the extracellular Ca^2+^ free buffer and replacement by a Ca^2+^ containing Ringer solution caused a maximal entry of Ca^2+^ through store operated ORAI1 Ca^2+^ influx channels (SOCE). All four ANO3 variants significantly reduced ER Ca^2+^ store filling, and this effect was caused by strongly attenuated SOCE (Fig. 1A). In contrast, wtANO3 only marginally affected SOCE and filling of the Ca^2+^ store. The most pronounced inhibition of SOCE and store filling was observed with V561L-ANO3 and S651N-ANO3 (Fig. 1B). Taken together, the pronounced inhibition of IP_3_ mediated Ca^2+^ release by ANO3 variants can be attributed to the strongly reduced ER Ca^2+^ store filling, due to compromised store-operated Ca^2+^ entry through ORAI1 channels [6].

**Fig. 2:**
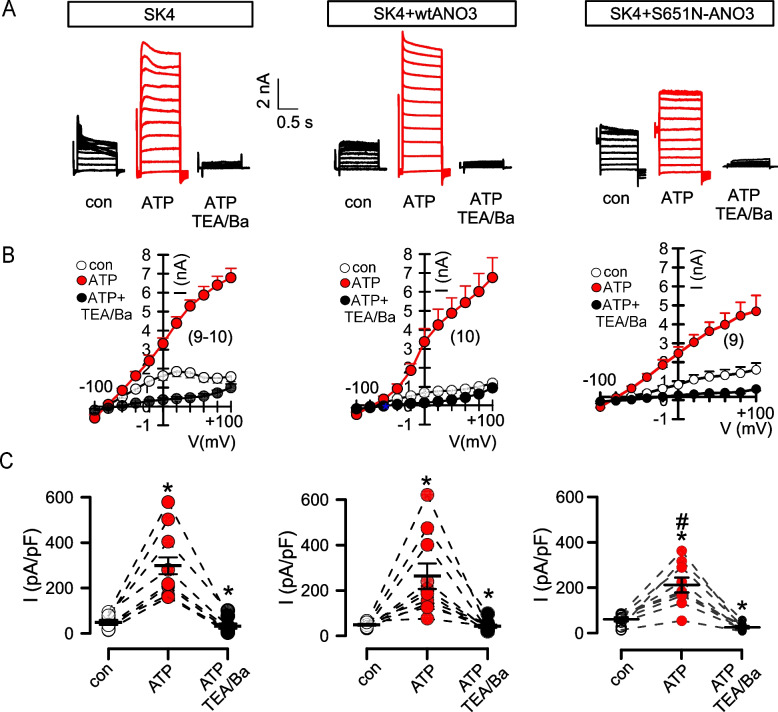
The S651N variant inhibits purinergic activation of SK4 K^+^ channels. **A** Original whole cell current overlays before and after stimulation of HEK293 cells expressing Ca^2+^-sensitive SK4 K^+^ channels with ATP (10 µM), and additional inhibition of SK4 channels by TEA/Ba (10 mM/5 mM). Activation of SK4 currents was not affected by coexpression of wt-ANO3 but was attenuated in the presence of S651N-ANO3. **B** Current–voltage (I/V) relationships corresponding to the whole cell currents shown in **A**. **C** Summary of the current densities corresponding to the data shown in **A** and **B**. Mean ± SEM (number of experiments). *significant increase by ATP (*p* < 0.05, paired t-test). ^#^reduced activation when compared to SK4 only (*p* < 0.05; ANOVA)

We previously reported that purinergic activation of K^+^ channels was inhibited in fibroblasts from a dystonia patient expressing S651N-ANO3 [6]. These findings suggest that inhibition of Ca^2+^ signals by ANO3 variants attenuates Ca^2+^-activation of small/intermediate conductance K^+^ channels of the KCNN family. To explore this further, we compared the effects of wtANO3 and S651N-ANO3 on ATP activation of K_Ca3.1_ (KCNN4, SK4). In HEK293 cells expressing K_Ca3.1_, ATP (10 µM) activated large K^+^ currents and hyperpolarized the membrane voltage, which was completely inhibited by TEA^+^/Ba^2+^. Coexpression of K_Ca3.1_ with S651N-ANO3 inhibited K^+^ current activation, while coexpression with wtANO3 had no effects (Fig. 2). These results suggest that inhibition of ATP-activated K^**+**^ currents by S651N-ANO3 is due to inhibition of K_Ca3.1_ channels and but not SLO1 (BK) channels, according to our previous data [6].

**Fig. 3:**
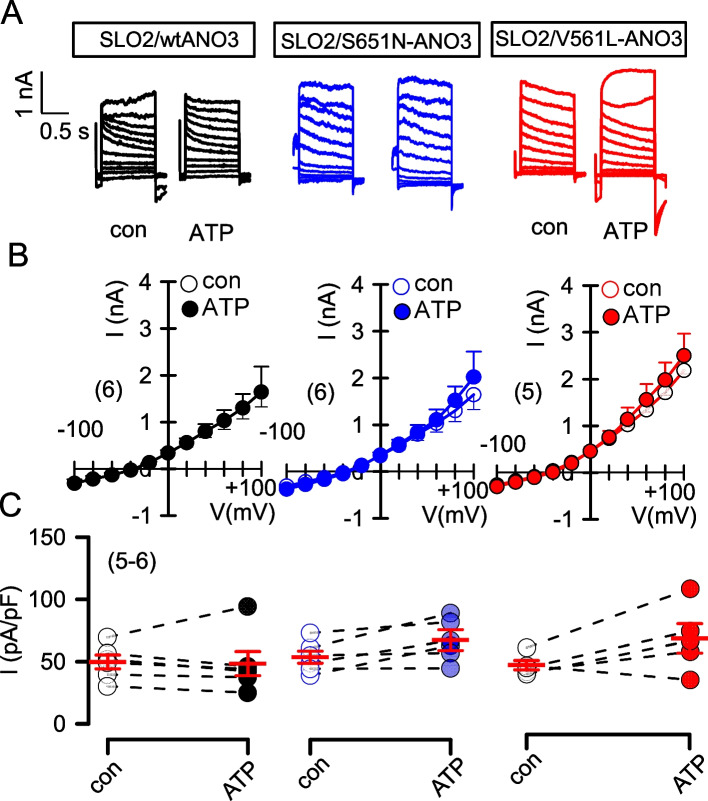
Currents produced by the K^+^ channel SLO2 are not activated by ATP and are not potentiated by wtANO3 or the ANO3 variants S651N and V561L. **A** Whole cell current overlays, **B** corresponding I/V curves, and **C** current densities in HEK293 cells overexpressing SLO2 together with wtANO3, S651N-ANO3 or V561L-ANO3. Cells were stimulated with ATP (10 µM). Mean ± SEM (number of experiments)

**Fig. 4:**
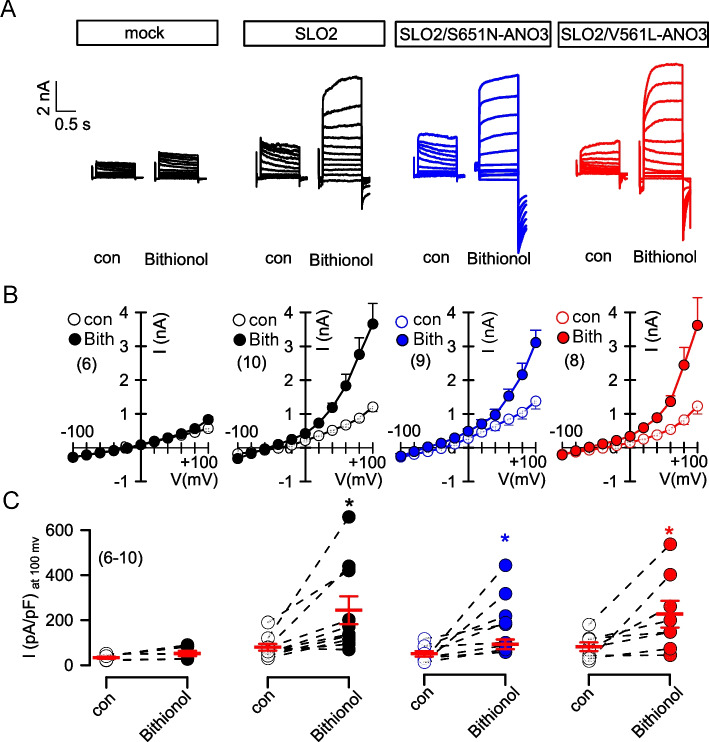
Activation of SLO2 by bithionol is not affected by the ANO3 variants S651N or V561L. **A** Whole cell current overlays, **B** corresponding I/V curves, and **C** current densities in HEK293 cells overexpressing SLO2 alone or together with S651N-ANO3 or V561L-ANO3. Mock-transfected cells served as controls. Cells were stimulated with the SLO2-activator bithionol (10 µM). ATP (10 µM). Mean ± SEM (number of experiments). *significant increase due to bithionol (*p* < 0.05, paired t-test)

SLO2 (SLACK, KCNT1, K_Na1.1_) is another regulator of neuronal excitability and was shown previously to be activated by ANO3 [10] [11]. It is unclear whether ATP activates SLO2 and, if so, whether activation of SLO2 is affected by coexpression of ANO3. Expression of SLO2 in HEK293 cells slightly enhanced basal whole cell currents (ΔI_Vc=+100 mV_ = 0.42 ± 0.21 nA; n = 5) and hyperpolarized membrane voltages (ΔVm = 9.2 ± 0.72 mV; n = 5). However, ATP did not further activate SLO2 currents in the absence or presence of wtANO3, S651N-ANO3, or V561L-ANO3 (Fig. 3). In contrast, bithionol (Bith), a known activator of SLO2 [12], strongly enhanced whole cell currents. Nevertheless, activation of SLO2 by bithionol was independent of coexpression with the ANO3 variants (Fig. 4). In summary, Ca^2+^/calmodulin-dependent activation of K_Ca3.1_ currents is inhibited by ANO3 variants, while we were unable to detect an effect of ANO3 on SLO1 or SLO2 K^+^ channels.

**Fig. 5:**
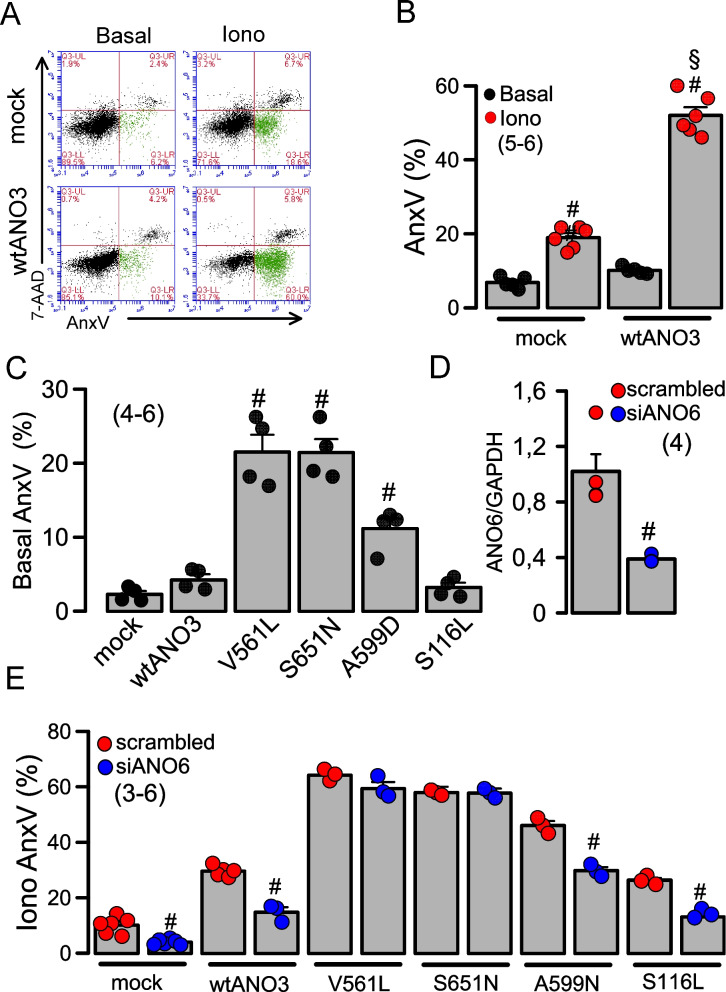
Basal and activated phospholipid scrambling is upregulated in ANO3 variants. **A** 4-quadrant dot blots of flow cytometry in mock transfected HEK293 cells or in cells expressing wtANO3. Cells were analyzed under control conditions (basal scrambling) and after incubation with ionomycin (Iono; 10 µM, 20 min), which strongly enhanced exposure of annexin V (AnxV). **B** Summary of % annexin V (AnxV) positive cells before and after stimulation with ionomycin. Increase of AnxV positive cells by ionomycin (^#^unpaired t-test (*p* < 0.05), which was augmented in wtANO3 expressing cells (^§^unpaired t-test, *p* < 0.05). **C** Phospholipid scrambling in nonstimulated (basal AnxV) cells expressing wtANO3 or ANO3 variants. ^#^significant difference when compared to wtANO3 (*p* < 0.05; ANOVA). **D** Summary of the knockdown of ANO6-expression by siRNA-ANO6. **E** Additive effects on ionomycin-induced scrambling (AnxV positivity) by endogenous ANO6 and overexpressed anoctamins (wtANO3, A599N-ANO3, and S1116L-ANO3). The pronounced scrambling activity of V561L-ANO3 and S651N-ANO3 fully compensated for the lack of ANO6 in siANO6 treated cells. Mean ± SEM (number of experiments). ^#^significant inhibition by siRNA-knockdown of ANO6-expression. (*p* < 0.05; unpaired t-test)

We previously reported the ability of ANO3 to scramble plasma membrane phospholipids [6]. Phospholipid scrambling, i.e. exposure of phosphatidylserine (PS) to the outer plasma membrane leaflet was demonstrated by fluorescent annexin V (AnxV), resulting in patchy staining of fibroblasts obtained from a dystonia patient heterozygous for S651N, but not of wild type fibroblasts (Additional file 1). Since this may be interpreted as enhanced basal phospholipid scramblase activity, we analyzed scrambling activity by ANO3 variants in more detail. When stimulated by Ca^2+^ ionophores such as ionomycin (Iono), ANO3 overexpressed in HEK293 cells strongly augmented AnxV-positivity (scrambling) in flow cytometry (Fig. 5A,B). Scrambling observed in mock-transfected cells is due to activation of ANO6, which is expressed endogenously in HEK293 cells [13].

Interestingly, basal scrambling, i.e. AnxV positivity in the absence of Iono, was enhanced in cells expressing the ANO3 variants V561L, S651N, and A599D, but not in mock-transfected cells or cells expressing wtANO3 or S116L-ANO3 (Fig. 5C). As expected, siRNA knockdown of endogenous ANO6 inhibited Iono-activated scrambling in mock-transfected cells and reduced scrambling in cells expressing wtANO3, A599N, and S116L, but not in cells expressing V561L and S651N (Fig. 5E,D). These results were further supported by propidium iodide (PI) staining in HEK293 cells expressing wtANO3 and the four different ANO3 variants. Propidium iodide uptake was monitored in nonstimulated cells, which was significantly enhanced in cells expressing V561L-ANO3 or S651N-ANO3, suggesting enhanced cell death by expression of these “severe” ANO3 variants (Additional file 2).

**Fig. 6:**
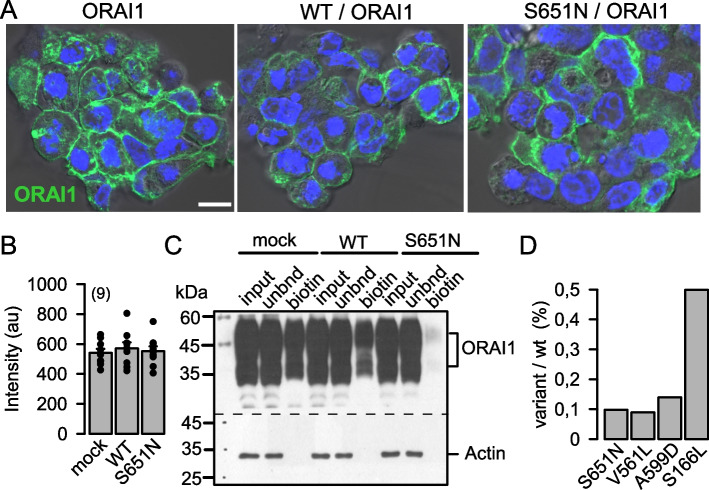
Plasma membrane expression of ORAI1 is inhibited in the presence of S651N-ANO3. **A** Overexpression of ORAI1 alone or coexpression of ORAI1 with wtANO3 (WT) or the ANO3 variant S651N. Bar = 20 µm. **B** Summary of ORAI1 membrane expression as detected by fluorescence intensity (arbitrary units; au) suggests no difference for the different conditions. Stainings were performed as triplicates. **C** Biotinylation of membrane proteins indicates strongly reduced expression of ORAI1 in the plasma membrane of ORAI1/S651N-ANO3 coexpressing cells (unbnd, unbound ORAI1 protein; biotin, biotinylated ORAI1 protein. Experiments were performed as triplicates. Mean ± SEM (number of experiments). **D** Ratios for ORAI1 biotinylation in the presence of wtANO3 or ANO3 variants (as shown in **C** and Additional file 3)

In addition, we investigated the molecular mechanism by which the disease-causing ANO3 variants induce the described functional changes. As the store-operated Ca^2+^ influx (SOCE) was largely inhibited ANO3 variants, we suggested a reduced expression of ORAI1 channels in the plasma membrane. Immunocytochemistry did not suggest a lack of overall expression of ORAI1 in cells expressing S651N-ANO3 (Fig. 6A,B). Because immunofluorescence does not discriminate between expression of ANO3 in the plasma membrane or submembranous compartments, we performed biotinylation of membrane proteins in cells expressing ORAI1 in the absence or presence of coexpressed wtANO3 or S651N-ANO3, and subsequent Western blotting of biotinylated ORAI1. In mock-transfected and wtANO3 coexpressing cells, ORAI1 was well detected in the biotinylated (biotin) fraction. In contrast, cells coexpressing S651N-ANO3 showed very little ORAI1 in the biotinylated fraction, suggesting low expression of ORAI1 in the plasma membrane (Fig. 6C,D). We performed similar experiments using the other ANO3 variants. Expression of V561L-ANO3 and A599D-ANO3 strongly inhibited ORAI1 biotinylation similar to S651N, while coexpression with S116L-ANO3 reduced biotinylation to a lesser degree (Additional file 3, Fig. 6D). Taken together, disease-causing ANO3 variants may lead to mislocalization of ORAI1 channels causing impaired Ca^2+^ signaling and attenuated activation of Ca^2+^ regulated K^+^ currents.

## Discussion

**Fig. 7:**
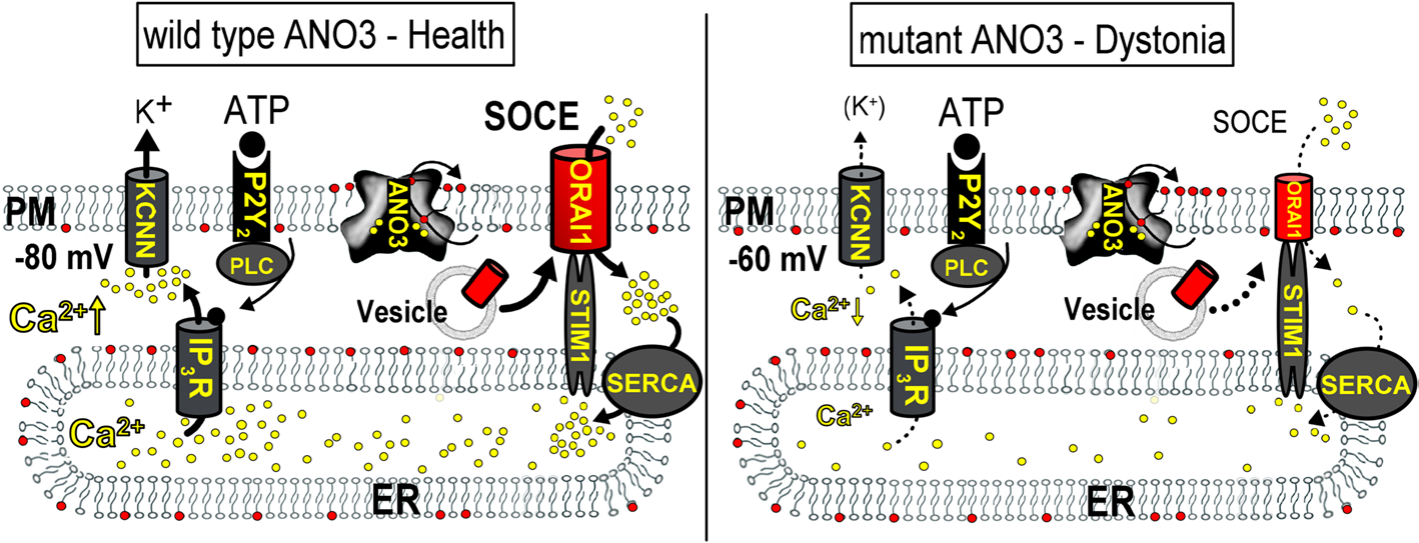
Proposed disease mechanism for dystonia caused by ANO3 variants. Wild type ANO3 expressed in the plasma membrane (PM) and in intracellular membranous compartments such as intracellular vesicles of striatal cells, allows for trafficking of ORAI1 Ca^2+^ channels to the PM, possibly by means of its phospholipid scramblase function. Thus, store-operated Ca^2+^ entry (SOCE) through ORAI1 activated through STIM1 causes proper filling of the endoplasmic reticulum (ER) Ca^2+^ store via the Ca^2+^ pump SERCA. Ca^2+^ release from the ER during activation of striatal cells causes activation of Ca^2+^ dependent KCNN K^+^ channels and repolarization of the membrane voltage to largely negative values (e.g. −80 mV). In striatal cells expressing mutant ANO3 variants, ORAI1 function, Ca^2+^ influx and ER store filling and activation of K^+^ channels are compromised causing reduced hyperpolarization (e.g. – 60 mV). This may lead to hyperexcitability and dystonia

In our previous study, we presented data on novel, i.e. previously unreported, and known ANO3 variants causing childhood-onset generalized dystonia. In fibroblasts from patients heterozygous for S116L or V561L, Ca^2+^-dependent activation of K^+^ currents was compromised due to attenuated Ca^2+^ signals [6]. The present data now provide a direct connection between mutations in ANO3, loss of plasma membrane expression of ORAI1, disturbed Ca^2+^ signaling, and dystonia. We show that store-operated Ca^2+^ influx via ORAI1 is profoundly reduced in cells expressing disease causing ANO3 variants, which reduces ER Ca^2+^ store filing and therefore attenuates receptor-mediated Ca^2+^ release by inositol 1,4,5-trisphosphate (IP_3_) and activation of K_Ca3.1_ channels (Fig. 7). ATP is known to activate members of the KCNN family of K^+^ channels (KCNN1-4), which are broadly expressed in the brain (https://www.proteinatlas.org/ENSG00000134343-ANO3/brain.). Upon other K^+^ channels, K_Ca3.1_ contributes to the so-called slow afterhyperpolarization required for repolarization of neurons, and also dependents on expression of the Ca^2+^-binding protein hippocalcin [14–17]. Like ANO3 variants, mutations in hippocalcin were shown to contribute to the appearance of dystonia [17, 18]. The role of K_Ca3.1_ in dystonia is poorly examined, however, genetic K_Ca3.1_ deficiency was shown to lead to locomotor hyperactivity in mice [19] and the activator of K_Ca3.1_, riluzole, was proposed as a therapy in an open-label pilot study and was also demonstrated to be beneficial in other movement disorders [20–24].

Our previous and the present data also demonstrate the scramblase activity of ANO3 [6] (Fig. 5). For ANO3 variants such as V561L, S651N, and A599D, we found a significant upregulation of phospholipid scrambling even under basal (nonstimulated) conditions, which suggested an enhanced Ca^2+^ sensitivity of these variants [6] (Fig. 5C). The present results now demonstrate strongly reduced plasma membrane expression of ORAI1 and largely attenuated store operated Ca^2+^ entry in the presence of ANO3 variants, leading to reduced filling of the ER Ca^2+^ store. Like the paralogous protein ANO10, also ANO3 is primarily an intracellular scramblase [5, 25]. ANO10 was shown to operate as an interorganelle regulator of endosomal sorting and loss of ANO10 function leads to impaired endosomal retrograde transport and neuromuscular function [26]. Although a role of ANO3 for intracellular traffic remains to be demonstrated, it could explain mistargeting of ORAI1 to intracellular compartments and, on the other hand, could lead to enhanced expression of ANO3 in the plasma membrane leading to enhanced scrambling and exposure of phosphatidylserine. However, enhanced membrane expression of endogenous ANO3 remains to be demonstrated and probably requires generation of transgenic animals.

Transgenic animals will also be required to determine phenotypic changes in true brain striatal cells. Observations in fibroblasts obtained from patients or experiments in overexpressing cells may provide initial pathogenic hints, however, the potential patho-mechanisms described here and previously [6], remain to be demonstrated in brain. Nevertheless, the impact of ANO3 variants on Ca^2+^ signaling were impressive and the potential regulation of protein expression in the plasma membrane by ANO3 may have unmasked similarities of ANO3 with other members of the anoctamin scramblase family like ANO6 and ANO10. Along with ANO6 and ANO10, ANO3 could regulate endosomal sorting, trafficking and exocytosis of membrane proteins and thus control the plasma membrane proteome [5, 26–28].

## Conclusions

Taken together, the present data provide potential pathogenic mechanisms underlying anoctamin-related dystonia. Dysfunction or damage of brain striatal neurons could be caused by abnormal Ca^2+^ signaling, ion currents, impaired repolarization, and enhanced phospholipid scrambling, which may even provide an eat-me signal and trigger phagocytosis by microglia [29, 30]. The functional data (ion currents, Ca^2+^ signaling, ORAI1 expression, phospholipid scrambling) assessed for the four different ANO3 variants suggest some correlation with the clinical phenotypes [6].

## Supplementary Information

Additional file 1. *Enhanced basal phospholipid scrambling in fibroblasts from a dystonia patient heterozygous for S651N.*Annexin V (AnxV) staining of phosphatidylserine (PS) exposed in the outer leaflet of the plasma membrane. In skin fibroblasts from a healthy volunteer, no PS could be stained (left panel), while in fibroblasts from a dystonia patient heterozygous for S651N, a spotted PS labeling was observed (right panel). Bar = 20 µm

Additional file 2. *Cell death assays suggest enhanced death of cells expressing dystonia-expressing variants of ANO3.* Cell death assays using propidium iodide (PI) uptake. HEK293 cells were transfected with wtANO3 or ANO3 variants known to cause dystonia. Propidium iodide uptake was monitored in nonstimulated cells under basal conditions. PI fluorescence intensity was significantly enhanced in cells expressing V561L-ANO3 or S651N-ANO3, suggesting enhanced cell death by expression of these ANO3 variants. Mean ± SEM, ^#^significantly different to wtANO3 (ANOVA).

Additional file 3. *Mutant ANO3 variants lead to reduced membrane expression of ANO3.* **A**) Biotinylation of membrane proteins suggests strongly reduced expression of ORAI1 in the plasma membrane of HEK293 cells coexpressing the ANO3 variant V561L-ANO3. **B**) A similar inhibition of ORAI1 biotinylation was observed by coexpression of the variant A599D-ANO3, while coexpression with S116L-ANO3 allowed for better membrane expression of ORAI1, which, however, still appeared to be reduced compared to cells coexpressing wtANO3. (unbnd, unbound ORAI1 protein; biotin, biotinylated ORAI1 protein).

Additional file 4.

## Data Availability

No datasets were generated or analysed during the current study.

## Abbreviations

ANO3: Anoctamin 3
K_Ca3.1_: Ca^2+^/calmodulin-activated K^+^ channels
ER: Endoplasmic reticulum
GPCRs: G-protein-coupled membrane receptors
IP3: Inositol trisphosphate
KCNN: Small Conductance Ca^2+^ activated K^+^ channel
ORAI1: Calcium Release-Activated Calcium Channel Protein 1
PtdSer: Phosphatidylserine
SERCA: Sarcoplasmic endoplasmic reticulum Ca^2+^ ATPase
SOCE: Store-operated Ca^2+^ entry
STIM1: Stromal Interaction Molecule 1
Wt: Wild-type
