# Supplementary material for: Dystonia caused by ANO3 variants is due to attenuated Ca2+ influx by ORAI1
Source: BMC Med. 2025 Jan 7;23:12. doi: 10.1186/s12916-024-03839-5 (PMC11707858; doi:10.1186/s12916-024-03839-5)
Supplement: Supplementary file 1 — Additional file 1. Enhanced basal phospholipid scrambling in fibroblasts from a dystonia patient heterozygous for S651N.Annexin V (AnxV) staining of phosphatidylserine (PS) exposed in the outer leaflet of the plasma membrane. In skin fibroblasts from a healthy volunteer, no PS could be stained (left panel), while in fibroblasts from a dystonia patient heterozygous for S651N, a spotted PS labeling was observed (right panel). Bar = 20 µm [file 12916_2024_3839_MOESM1_ESM.pdf]

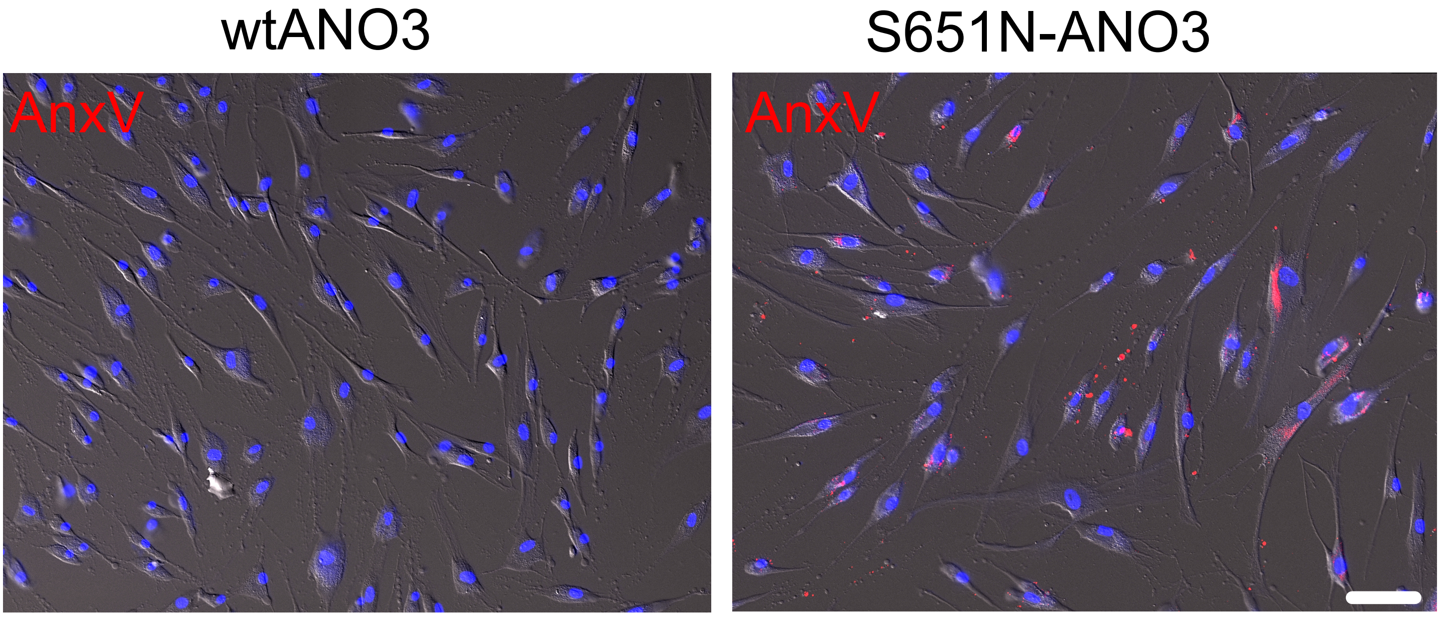

Additional file 1. *Enhanced basal phospholipid scrambling in fibroblasts from a dystonia patient heterozygous for S651N.* Annexin V (AnxV) staining of phosphatidylserine (PS) exposed in the outer leaflet of the plasma membrane. In skin fibroblasts from a healthy volunteer, no PS could be stained (left panel), while in fibroblasts from a dystonia patient heterozygous for S651N, a spotted PS labeling was observed (right panel). Bar = 20 µm.
