# Supplementary material for: Dystonia caused by ANO3 variants is due to attenuated Ca2+ influx by ORAI1
Source: BMC Med. 2025 Jan 7;23:12. doi: 10.1186/s12916-024-03839-5 (PMC11707858; doi:10.1186/s12916-024-03839-5)
Supplement: Supplementary file 2 — Additional file 2. Cell death assays suggest enhanced death of cells expressing dystonia-expressing variants of ANO3. Cell death assays using propidium iodide (PI) uptake. HEK293 cells were transfected with wtANO3 or ANO3 variants known to cause dystonia. Propidium iodide uptake was monitored in nonstimulated cells under basal conditions. PI fluorescence intensity was significantly enhanced in cells expressing V561L-ANO3 or S651N-ANO3, suggesting enhanced cell death by expression of these ANO3 variants. Mean ± SEM, #significantly different to wtANO3 (ANOVA). [file 12916_2024_3839_MOESM2_ESM.pdf]

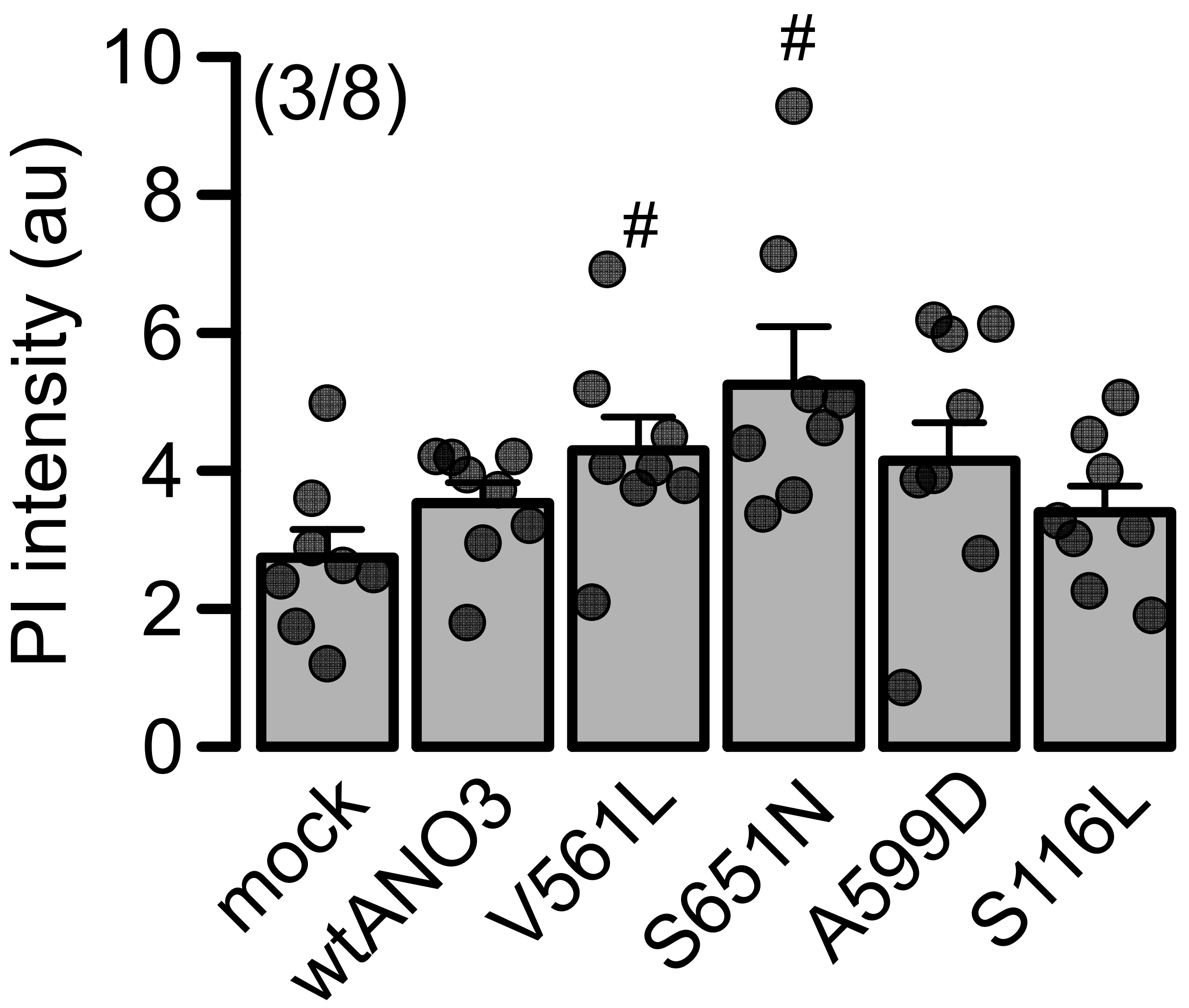

Additional file 2. *Cell death assays suggest enhanced death of cells expressing dystonia-expressing variants of ANO3.* Cell death assays using propidium iodide (PI) uptake. HEK293 cells were transfected with wtANO3 or ANO3 variants known to cause dystonia. Propidium iodide uptake was monitored in nonstimulated cells under basal conditions. PI fluorescence intensity was significantly enhanced in cells expressing V561L-ANO3 or S651N-ANO3, suggesting enhanced cell death by expression of these ANO3 variants. Mean ± SEM, #significantly different to wtANO3 (ANOVA).
