# Supplementary material for: Dystonia caused by ANO3 variants is due to attenuated Ca2+ influx by ORAI1
Source: BMC Med. 2025 Jan 7;23:12. doi: 10.1186/s12916-024-03839-5 (PMC11707858; doi:10.1186/s12916-024-03839-5)
Supplement: Supplementary file 3 — Additional file 3. Mutant ANO3 variants lead to reduced membrane expression of ANO3. A) Biotinylation of membrane proteins suggests strongly reduced expression of ORAI1 in the plasma membrane of HEK293 cells coexpressing the ANO3 variant V561L-ANO3. B) A similar inhibition of ORAI1 biotinylation was observed by coexpression of the variant A599D-ANO3, while coexpression with S116L-ANO3 allowed for better membrane expression of ORAI1, which, however, still appeared to be reduced compared to cells coexpressing wtANO3. (unbnd, unbound ORAI1 protein; biotin, biotinylated ORAI1 protein). [file 12916_2024_3839_MOESM3_ESM.pdf]

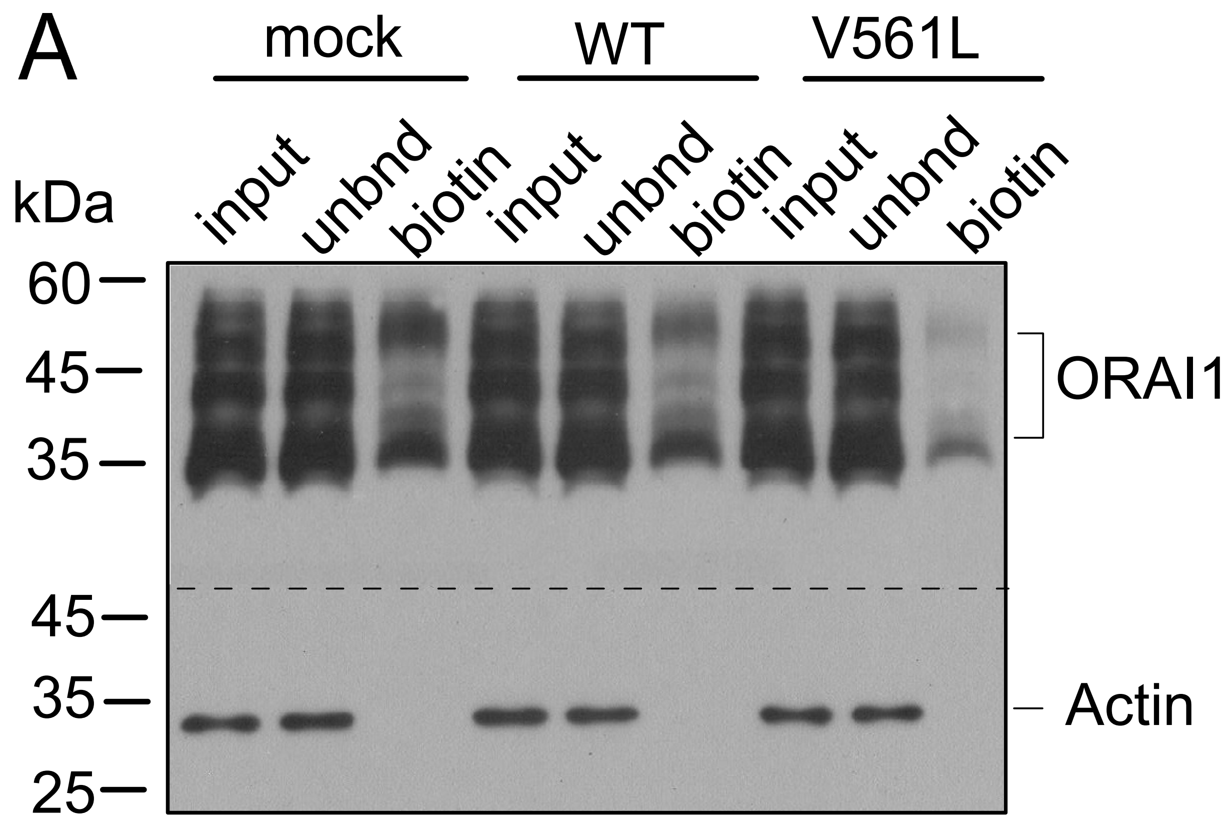

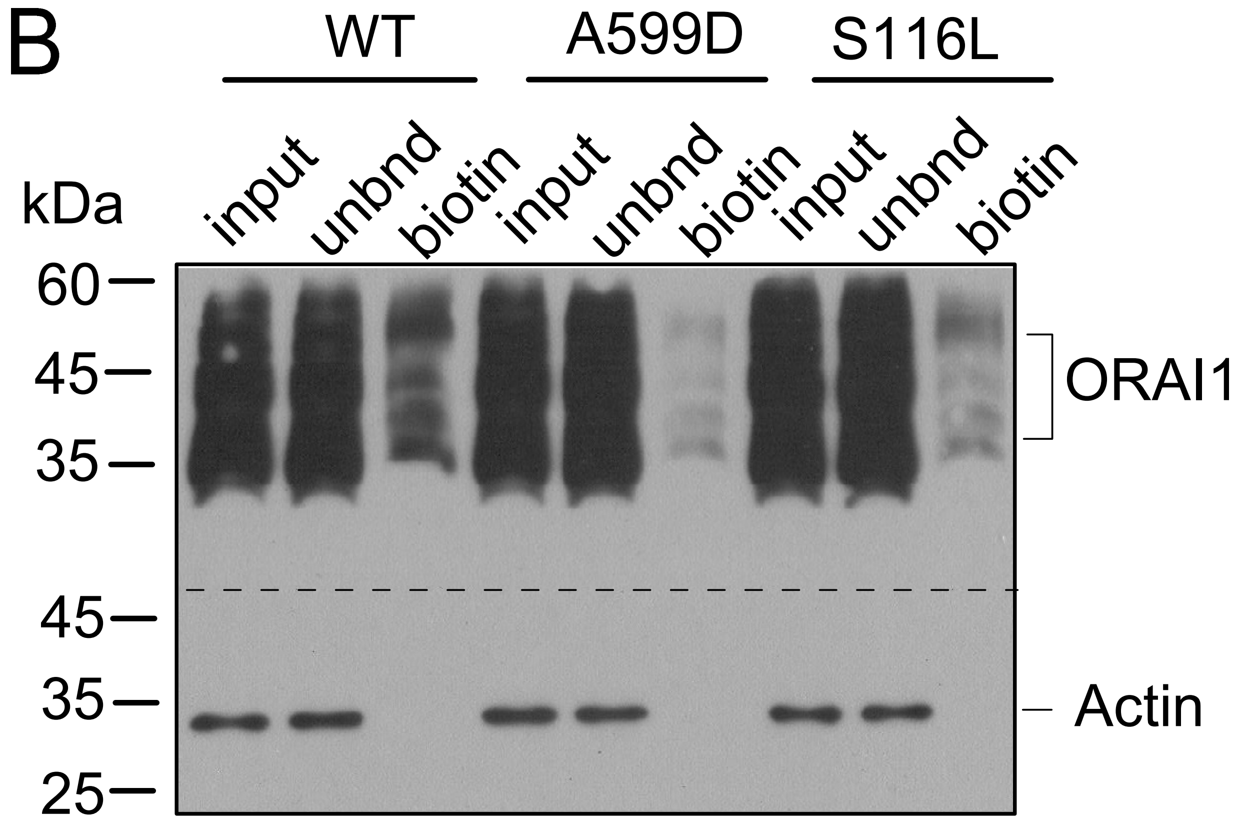

Additional file 3. *Mutant ANO3 variants lead to reduced membrane expression of ANO3.* **A**) Biotinylation of membrane proteins suggests strongly reduced expression of ORAI1 in the plasma membrane of HEK293 cells coexpressing the ANO3 variant V561L-ANO3. **B**) A similar inhibition of ORAI1 biotinylation was observed by coexpression of the variant A599D-ANO3, while coexpression with S116L-ANO3 allowed for better membrane expression of ORAI1, which, however, still appeared to be reduced compared to cells coexpressing wtANO3. (unbnd, unbound ORAI1 protein; biotin, biotinylated ORAI1 protein).
