# Supplementary figures and images for: Dystonia caused by ANO3 variants is due to attenuated Ca2+ influx by ORAI1
Source: BMC Med. 2025 Jan 7;23:12. doi: 10.1186/s12916-024-03839-5 (PMC11707858; doi:10.1186/s12916-024-03839-5)

# Uncropped blots

Fig. 6C

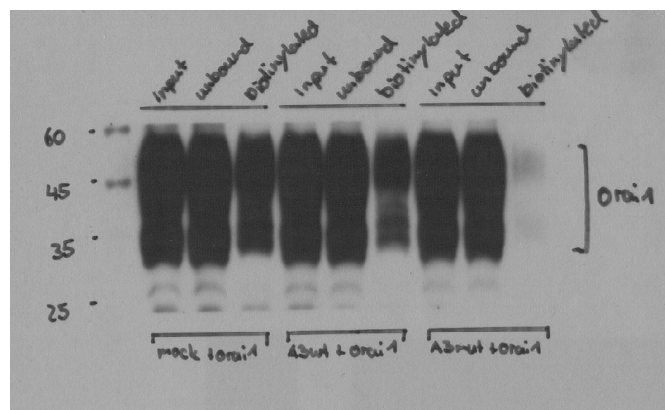

Supp. Fig. 3A

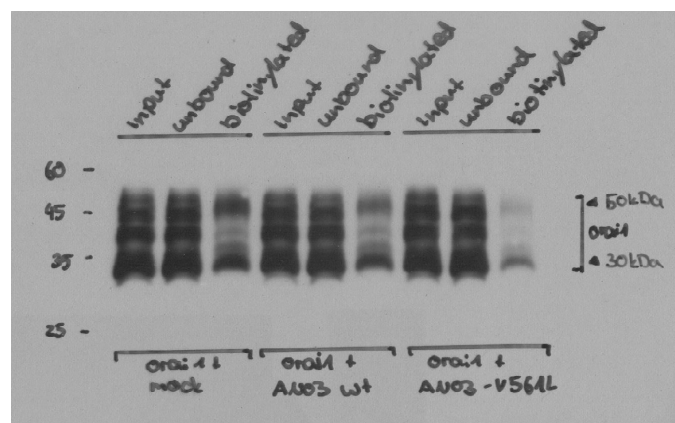

Supp. Fig. 3B

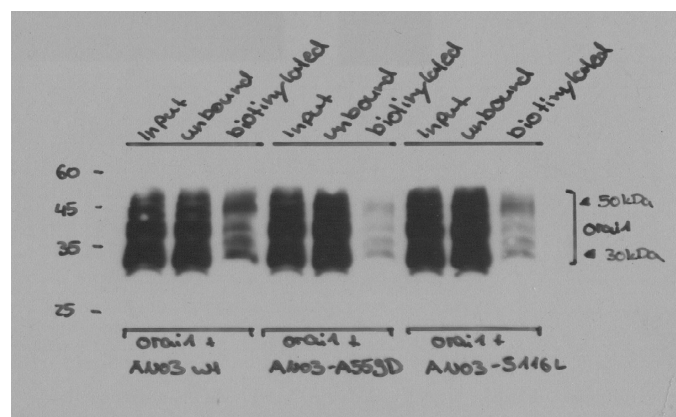

Supplement: Supplementary file 4 — Additional file 4. [file 12916_2024_3839_MOESM4_ESM.pdf]
